# Sex differences in ST-segment elevation myocardial infarction patients treated by primary percutaneous intervention

**DOI:** 10.1136/openhrt-2024-002831

**Published:** 2025-01-04

**Authors:** Selma T Cook, Laure Allemann, Malica Cook, Diego A Arroyo, Thais Pittet, Pascal Meier, Mario Togni, Amel Brahim-Mathiron, Serban Puricel, Stéphane Cook

**Affiliations:** 1 Cardiology, University Hospital Fribourg, Fribourg, Switzerland

**Keywords:** Acute Coronary Syndrome, Atherosclerosis, Clinical Competence

## Abstract

**Introduction:**

The impact of sex on coronary artery disease prognosis is debated. It has been postulated that women receive less prompt treatment compared with men, potentially adversely affecting their prognosis by significantly increasing the risk of morbidity and mortality. We aim to investigate the influence of sex on the timing and clinical outcomes of ST-segment elevation myocardial infarction (STEMI) patients using a controlled Swiss registry.

**Methods and results:**

Based on the Fribourg STEMI Fast Track Registry, 1177 patients (288 women, 889 men) with >12 months clinical follow-up were selected. Women had longer first medical contact to reperfusion times (1.31 (1.14–2.00) vs 1.27 (1.09–1.54) hours, p=0.035) but similar total ischaemic times (3.04 (2.15–4.50) vs 2.56 (2.07–4.38) hours, p=0.064). Men had higher rates of diabetes, smoking and dyslipidaemia, while women had higher hypertension and renal insufficiency rates. No significant sex differences in clinical outcomes were observed at 1-year and 5-year follow-ups.

**Discussion:**

The study found sex differences in patient profiles and minor treatment delays for women, which did not significantly affect outcomes. Efforts to improve sex equity in STEMI care are effective, as no significant outcome differences were observed. Disparities are more related to patient characteristics than sex.

**Conclusion:**

Despite slight delays and different risk profiles for women with STEMI, clinical outcomes are similar between sexes. Ongoing efforts are needed to ensure sex equity in acute coronary syndrome management.

**Trial registration number:**

NCT04185285.

**WHAT IS ALREADY KNOWN ON THIS TOPIC**

- Women are often managed later than men in acute coronary syndromes. Delays in care for women have been observed, but it remains unclear whether this applies universally, including in unselected ST-segment elevation myocardial infarction (STEMI) populations.

**WHAT THIS STUDY ADDS**

- In this cohort, women experienced delayed management, with first medical contact to revascularisation time extended by 2.4 min. This aligns with findings from similar studies. Slight differences in clinical profiles, such as age, may partly explain these delays. However, the primary driver appears to be stereotypes about patients (heart attacks occur in men over 50) rather than issues of societal sex equity. Despite delays, long-term clinical outcomes for women were similar to men.

**HOW THIS STUDY MIGHT AFFECT RESEARCH, PRACTICE OR POLICY**

- Addressing stereotypes that marginalise certain patient groups, such as women and younger individuals in STEMI, remains a critical challenge. Encouragingly, the gender gap in management appears to be narrowing, likely due to improved awareness among emergency teams. Recent gender guidelines highlight education as a priority for further progress in equitable care.

## Introduction

 The impact of sex on the prognosis of coronary artery diseases remains a subject of debate. Numerous studies have indicated sex-related differences in the clinical presentation, management and outcomes of ST-segment elevation myocardial infarction (STEMI) patients. These disparities encompass delays in hospital admission, atypical symptoms in women leading to diagnostic challenges, treatment response variations and variations in the prevalence of specific comorbidities.

Research has demonstrated that women may present with atypical myocardial infarction (MI) symptoms, contributing to delays in medical intervention. Additionally, observations suggest that women may be less likely to receive certain recommended treatments, such as early coronary revascularisation (REVASC), compared with their male counterparts.^1^,^3^

Concerning 1-year survival rates, some studies suggest that women with STEMI experience less favourable outcomes than men. However, these differences are likely influenced by variables such as age, comorbidities and other clinical factors. A comprehensive meta-analysis of 35 studies, including 18 555 women and 49 981 men undergoing primary percutaneous coronary intervention (PPCI) for STEMI, revealed that women had nearly twice the risk of in-hospital all-cause mortality and 1.5 times the risk of 1-year all-cause mortality compared with men. Yet, when adjusted for baseline cardiovascular risk factors and clinical profiles, the sex differences in outcomes were substantially reduced.^4^

Recent insurance data-driven research has highlighted an alarming trend in Switzerland, showing that women receive suboptimal management for acute coronary syndromes (ACSs), with significant delays in treatment initiation.^5^ This finding is particularly concerning as it contrasts with the growing awareness among healthcare professionals of sex disparities and the advancements in diagnostic tests and treatment protocols for ACSs. In light of these surprising results, and given the high prognostic stakes associated with STEMI incidents, we have decided to challenge these conclusions using a controlled Swiss registry. Our study aims to investigate the influence of sex on the timing and clinical endpoints of patients presenting with STEMI, to ensure that our treatment protocols effectively address these critical disparities.

## Methods

### Patient and public involvement

The Gender Evaluation in Myocardial Time Examination for Cardiac Acute Response is based on the prospective Fribourg STEMI Fast Track Registry, which has been collecting data since 2008. All patients with a suspected diagnosis of STEMI referred to the University Hospital Fribourg via the local STEMI network were eligible for enrolment. Patients were excluded if the definite diagnosis was not STEMI, they were unwilling or unable to provide written informed consent or they were unwilling to participate in follow-up. Baseline patient and procedural characteristics, as well as time delays, were collected on enrolment. Clinical follow-up was performed by phone or clinic visit at 1 month, 1 year and then yearly until 5 years. The registry is registered in ClinicalTrials.gov, number NCT04185285.

For this interim analysis, we included all patients enrolled from August 2008 to December 2023 whose first medical contact (FMC) time was known. All other patients were excluded from the present analysis.

### STEMI-network Fribourg

The STEMI Fast Track network Fribourg, established in 2008, provides a 24-hour PPCI service. The network aims to transfer confirmed STEMI patients directly to the catheterisation laboratory at Fribourg Hospital, bypassing the emergency department to ensure timely medical treatment. From 2008 to 2009, clopidogrel was used alongside aspirin as a second antiplatelet agent. Starting in 2009, ticagrelor and prasugrel became available and were preferred over clopidogrel. Patients were enrolled between August 2008 and December 2023. PPCI was performed according to the 2008 recommendations of the European Society of Cardiology (ESC) guidelines for STEMI.

### Clinical endpoints

The objective of the present study is to identify variables that significantly influence the delay between the FMC and REVASC and to evaluate the impact of this delay on subsequent clinical outcomes in our population. The primary endpoint was the time from FMC to REVASC. Secondary endpoints included survival free from major adverse cardiac events (MACE—composite of cardiac death (CD), non-fatal MI and any unplanned REVASC), all-cause mortality and the individual components of the primary endpoint.

### Definitions

Pain was defined as the moment of symptom onset. FMC was defined as the moment a medical professional initially assesses the patient. REVASC was defined as the moment the guidewire passes through the culprit lesion. Dyslipidaemia was defined according to the Adult Treatment Panel III.^6^ Death was classified as either cardiac or non-cardiac, according to the Academic Research Consortium (ARC) definition.^7^ Deaths that could not be classified were considered cardiac. Unplanned REVASC was defined as any repeat percutaneous or surgical REVASC, regardless of location. Stent thrombosis was classified as either definite or probable and defined according to the ARC definitions.

### Statistical analysis

Analyses were performed using SPSS software V.26.0 (SPSS, Chicago, Illinois, USA) and STATA V.14 MP (StataCorp, College Station, Texas, USA). Continuous variables are expressed as mean±SD or median with IQR. Categorical variables are expressed as counts and percentages. Histograms were used to assess normal distribution for continuous variables.

In a univariate analysis, baseline characteristics, procedural details and clinical outcomes were compared between women and men using χ^2^ test for categorical variables, unpaired t-tests for continuous variables with a normal distribution and non-parametric tests such as the Wilcoxon rank-sum test or the Mann-Whitney U test for continuous variables with a non-Gaussian distribution.

For correlation of normally distributed variables, the Pearson product-moment correlation coefficient was calculated. For variables with a non-Gaussian distribution, Spearman’s rank correlation coefficient was computed.

Crude estimates of clinical outcomes were assessed using the Kaplan-Meier method. Cox regression was used to adjust for baseline imbalances between sexes. In order to identify independent predictors of the occurrence of MACE, a statistical model was computed using Cox proportional hazards and stepwise backwards elimination with elimination criteria of >0.25 and retention criteria of <0.10.

## Results

A total of 1209 patients were admitted with STEMI, of whom 1177 met the inclusion and exclusion criteria and were included in the present study (288 women, 889 men). Baseline characteristics are presented in table 1. All patients were considered for PPCI. No patient received thrombolysis. Mean age was 62.8±12.6 years (p=0.11).

**Table 1:** Baseline patient characteristics

	All (n=1177)	Women (n=288)	Men (n=889)	P value
Age (years), mean±SD	62.75±12.61	67.31±13.03	61.27±12.11	0.107
BMI (kg/m^2^), mean±SD	26.88±4.30	25.97±4.84	27.18±4.07	0.003
Diabetes mellitus, n (%)	190 (16.1)	35 (12.2)	155 (17.4)	0.034
Insulin dependant	35 (3.0)	12 (4.2)	23 (2.6)	0.167
Smoking, n (%)	686 (58.3)	148 (51.4)	538 (60.5)	0.007
Arterial hypertension, n (%)	584 (49.6)	176 (61.1)	408 (45.9)	<0.001
Dyslipidaemia, n (%)	582 (49.4)	128 (44.4)	454 (51.1)	0.049
Family history, n (%)	233 (19.8)	47 (16.3)	186 (20.9)	0.090
Previous MI, n (%)	116 (9.9)	13 (4.5)	103 (11.6)	<0.001
Previous PCI, n (%)	153 (13.0)	19 (6.6)	134 (15.1)	<0.001
Previous CABG, n (%)	19 (1.6)	2 (0.7)	17 (1.9)	0.187
Renal failure, n (%)	18 (1.5)	10 (3.5)	8 (0.9)	0.004
Admitted during working hours, n (%)	632 (53.7)	155 (53.8)	477 (53.7)	1.000
Cardiogenic shock, n (%)	106 (9.0)	23 (8.0)	83 (9.3)	0.554
Coronary artery disease				
Single vessel, n (%)	440 (37.4)	122 (42.4)	318 (35.8)	0.050
Two vessels, n (%)	382 (32.5)	93 (32.3)	289 (32.5)	1.000
>two, n (%)	354 (30.1)	72 (25.0)	282 (31.7)	0.032
Culprit lesion				
Left main, n (%)	16 (1.4)	2 (0.7)	14 (1.6)	0.383
LAD, n (%)	525 (44.6)	144 (50.0)	381 (42.9)	0.035
LCX, n (%)	158 (13.4)	39 (13.5)	119 (13.4)	0.921
RCA, n (%)	470 (39.9)	102 (35.4)	368 (41.4)	0.072
Multivessel index				
Single vessel PCI, n (%)	746 (63.4)	196 (67.9)	550 (61.8)	0.058
Multivessel PCI, n (%)	431 (36.6)	92 (32.1)	339 (38.2)	
Haemodynamic support				
IABP, n (%)	49 (4.2)	12 (4.2)	37 (4.1)	0.977
ECMO, n (%)	6 (0.5)	1 (0.3)	5 (0.6)	
pVAD, n (%)	8 (0.7)	2 (0.7)	6 (0.7)	
No assistance, n (%)	1114 (94.6)	273 (94.8)	841 (94.6)	
Provenance				
Walk-in to ER of PCI centre, n (%)	258 (21.9)	50 (17.4)	208 (23.4)	0.033
Transfer from another hospital, n (%)	345 (29.3)	71 (24.7)	274 (30.8)	0.053
Referral from GP, n (%)	79 (6.7)	15 (5.2)	64 (7.2)	0.279
EMS, n (%)	473 (40.2)	142 (49.3)	331 (37.2)	<0.001
Other or unknown, n (%)	22 (1.9)	10 (3.5)	12 (1.4)	0.021

BMI, body mass index; CABG, coronary artery bypass graft; ECMO, extracorporeal membrane oxygenation; EMS, emergency medical servicesER, emergency room; GP, general practitioner; IABP, intra-aortic balloon pump; LAD, left anterior descending artery; LCX, left circumflex artery; MI, myocardial infarction; PCI, percutaneous coronary intervention; pVAD, percutaneous ventricular assist device; RCA, right coronary artery

### Delays

**Figure 1:**
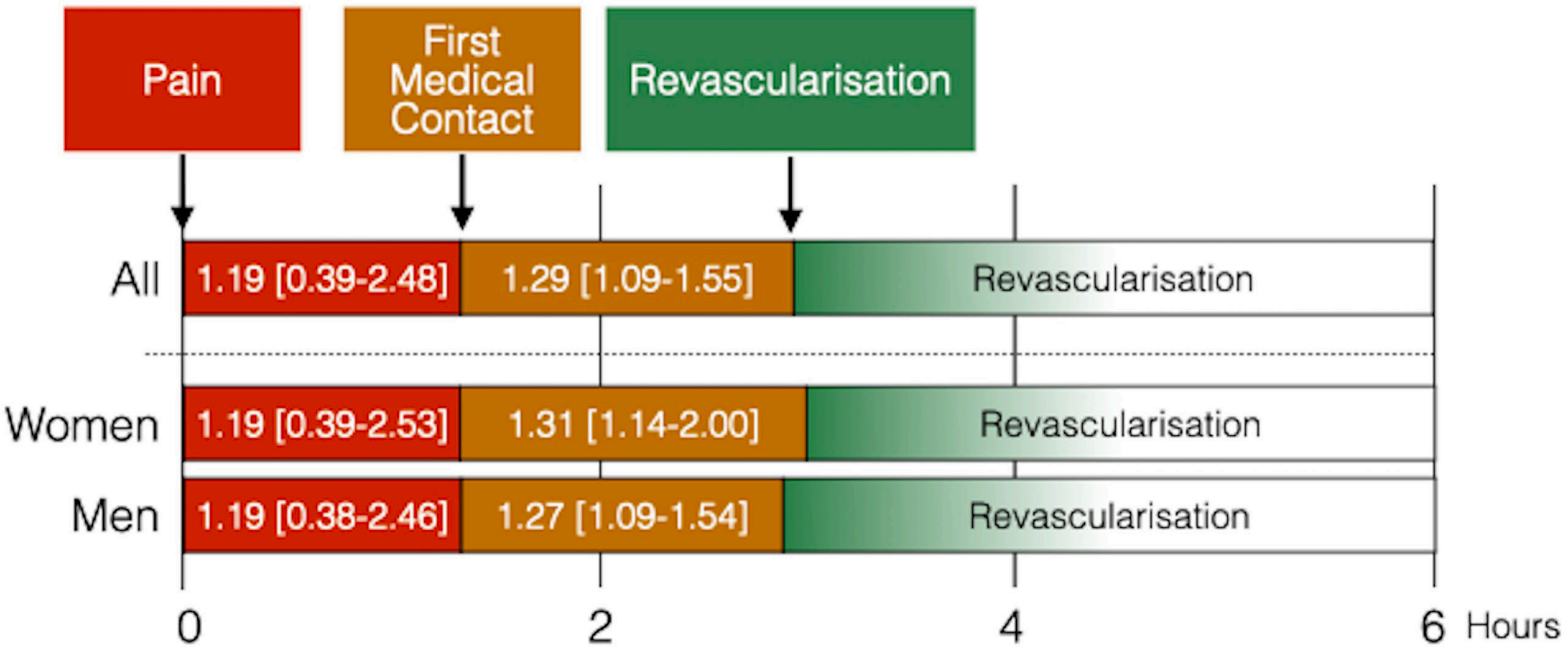
Illustration of time delays. Time delays from pain onset to first medical contact (FMC) (red) and from FMC to revascularisation (REVASC) (orange) and REVASC (green). Median times (in hours) with IQRs (25–75) are shown for all patients, women and men.

There was no significant difference between men and women in ‘pain to FMC’ time (median (IQR: 25–75); women: 1.19 (0.39–2.53) hours vs men: 1.19 (0.38–2.46) hours, p=0.562). Overall, the ‘FMC to reperfusion’ time (FMC-R: median (IQR: 25–75)) was 1.29 (1.09–1.55) hours, with 1.31 (1.14–2.00) hours for women and 1.27 (1.09–1.54) hours for men, indicating a statistically significant difference (p=0.035). Median ‘total ischaemic’ time was similar between sexes (pain to reperfusion: median (IQR 25–75); women: 3.04 (2.15–4.50) hours vs men: 2.56 (2.07–4.38) hours, p=0.064) (table 2). Figure 1 illustrates the time delays from pain onset to FMC and from FMC to REVASC. The median times (in hours) with IQRs (25–75) are shown for all patients, women and men.

**Table 2:** Provenance and delays

	All (n=1177)	Women (n=288)	Men (n=889)	P value
Pain to FMC, hours. Median (IQR 25–75)	1.19 (0.39–2.48)	1.19 (0.39–2.53)	1.19 (0.38–2.46)	0.562
FMC to REVASC, hours. Median (IQR 25–75)	1.29 (1.09–1.55)	1.31 (1.14–2.00)	1.27 (1.09–1.54)	0.035
Pain to REVASC (total ischaemic time), hours. Median (IQR 25–75)	2.59 (2.07–4.40)	3.04 (2.15–4.50)	2.56 (2.07–4.38)	0.064

FMC, first medical contact; REVASC, revascularisation

### Cardiovascular risk factors

The results of our study reveal significant differences in cardiovascular risk factors between men and women. Among men, we observed a higher prevalence of diabetes mellitus, with 17.4% (n=155) compared with 12.2% (n=35, p=0.034) for women. Similarly, smoking was significantly more common among men, with a prevalence of 60.5% (n=538) compared with 51.4% (n=148, p=0.007) for women. Men also exhibited a higher prevalence of dyslipidaemia, with 51.1% (n=454) compared with 44.4% (n=128, p=0.049) for women. Additionally, it was observed that men generally have a higher body mass index than women (women: 26.0±4.8 vs men: 27.2±4.1, p=0.003).

However, no significant difference was observed in the prevalence of a family history between men and women, with an average prevalence of 19.8% (n=233, p=0.09). Among women, we found a higher prevalence of high blood pressure, with 61.1% (n=176) of women compared with 45.9% (n=408, p<0.001) of men. Finally, women exhibited a higher prevalence of renal insufficiency at 3.5% (n=10) compared with a prevalence of 0.9% (n=8, p=0.004) in men.

### Medical history

For personal medical history, men had a higher prevalence of previous MI, with 11.6% (n=103) compared with 4.5% (n=13, p<0.001) among women. Men also showed a higher prevalence of previous PCI, at 15.1% (n=134), whereas women had 6.6% (n=19, p<0.001).

### Coronary artery disease and lesion

No disparity was observed in the localisation of the lesions. A total of 2267 lesions were treated in 1177 patients. No significant difference in prevalence was observed between single-vessel (all: n=440, 37.4%, p=0.05) and two-vessel (all: n=382, 32.5%, p=1.00) lesions. However, for lesions involving more than two vessels, a prevalence of 31.7% (n=282) was recorded in men, compared with 25.0% (n=72, p=0.032) in women. There was a trend (p=0.06) towards more multivessel PCI in men (n=339, 38.2%) compared with women (n=92, 32.1%). No significant difference was observed in the use of haemodynamic support systems between the sexes (intra-aortic balloon pump: 4.1% vs 4.2%, percutaneous ventricular assist device: 0.7% vs 0.7%, extracorporeal membrane oxygenation: 0.3% vs 0.6%; p=0.98) (table 1).

### Clinical outcomes

**Figure 2:**
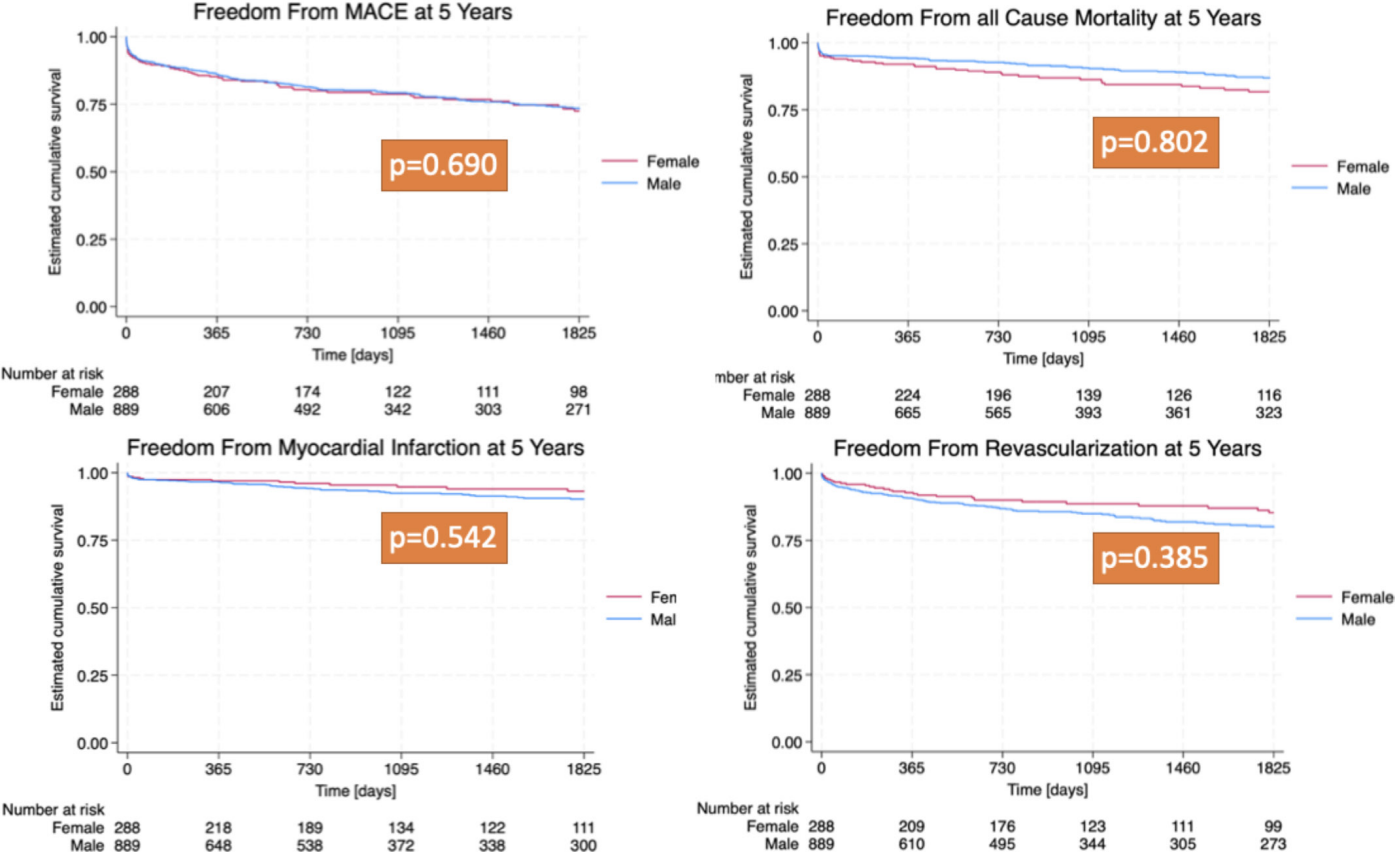
Kaplan-Meier survival curves—all-cause mortality and major adverse cardiac events (MACE). Kaplan-Meier survival curves show non-significant differences between women (red) and men (blue) for major adverse cardiovascular events (p=0.690), all-cause mortality (p=0.802), myocardial infarction (p=0.542) and revascularisation (p=0.385) at 5 years.

The clinical outcomes are summarised in table 3. At 1 year, no statistically significant disparity was observed in the incidence of total major adverse cardiovascular events (MACE) between the male and female cohorts, with a total of 149 cases (12.7%, p=0.82). Furthermore, there was no discernible difference between the sexes in terms of CD, non-fatal MI, repeat REVASC and all-cause mortality within the first year. Over a 5-year follow-up period, no statistically significant variance was noted in total MACE occurrences between the sexes. The rates of CD, non-fatal MI, repeat REVASC and all-cause mortality also showed no significant differences between men and women over this period. Figure 2 illustrates the Kaplan-Meier curves of the different MACE for both sexes.

**Table 3:** Primary clinical endpoint at 1 and 5 years according to sex

	All (n=1177)	Women (n=288)	Men (n=889)	P value
**1-year MACE, n(%)**	**149(12.7)**	**39(13.5)**	**110(12.4)**	**0.823**
Cardiac death (CD), n (%)	56 (4.8)	20 (6.9)	36 (4.0)	0.307
Non-fatal MI, n (%)	33 (2.8)	7 (2.4)	26 (2.9)	0.539
Repeat revascularisation (REVASC), n (%)	89 (7.6)	18 (6.3)	71 (8.0)	0.306
Stroke, n (%)	27 (2.3)	9 (3.1)	18 (2.0)	0.610
Bleeding, n (%)	58 (4.9)	21 (7.3)	37 (4.2)	0.576
Other death, n (%)	12 (1.0)	2 (0.7)	10 (1.1)	0.525
All death, n (%)	68 (5.7)	22 (7.6)	46 (5.2)	0.121
**5-year MACE, n(%)**	**239(20.3)**	**63(21.9)**	**176(19.8)**	**0.690**
CD, n (%)	88 (7.5)	31 (10.8)	57 (6.4)	0.210
Non-fatal MI, n (%)	72 (6.1)	14 (4.9)	58 (6.5)	0.542
Repeat REVASC, n (%)	152 (12.9)	30 (10.4)	122 (13.7)	0.385
Stroke, n (%)	51 (4.3)	13 (4.5)	38 (4.3)	0.890
Bleeding, n (%)	78 (6.6)	24 (8.3)	54 (6.1)	0.622
Other death, n (%)	37 (3.1)	10 (3.5)	27 (3.0)	0.431
All death, n (%)	125 (10.6)	41 (14.2)	84 (9.4)	0.802

MACE, major adverse cardiac event; MI, myocardial infarction

## Discussion

Our study aimed to explore the impact of sex on the effectiveness of STEMI management within our network. We discovered that: (1) patient profiles differ significantly by sex, (2) there is a slight discrepancy in waiting times, with women experiencing an average delay from FMC to REVASC of 2.4 min longer than men, and (3) clinical outcomes during follow-up are similar between sexes.

### Divergent patient profile

The study unveiled distinct differences between female and male profiles. Women typically present with advanced age, higher instances of hypertension and renal failure, yet have lower smoking rates than men. These findings align with existing research. For example, women are often older at the time of their first MI, with a difference of 7 years in US national statistics.^8^ In the Dutch ACS registry, women made 10% of patients under 65 years but 55% of those over 85 years.^9^ High blood pressure and insulin-requiring diabetes mellitus were identified in the INTERHEART Study.^10^ Specifically, these authors concluded that high blood pressure was more strongly associated with MI in women than in men and that eliminating high blood pressure could reduce the risk of heart attack by 36%. In our registry, the incidence of diabetes mellitus was similar between sexes, with a non-significant increase in the insulin-requiring subtype.^11^,^13^

### Equal time to treatment

Despite historical sex disparities leading to delayed diagnosis and treatment in women,^14 15^ our current data show that, due to heightened medical awareness, wait times have generally equalised. However, women still experience an additional delay of approximately 2.4 min compared with men. While statistically significant, this delay represents only 1% of the total ischaemic time and is unlikely to influence the prognosis. The medical community has become more vigilant in identifying MIs in women, who may present with atypical symptoms compared with men. Yet, as demonstrated by Rubini Gimenez *et al*^16^ in their European multicentre study, the differences in sex-specific chest pain characteristics are less pronounced than previously thought. This study, involving 2495 patients with a third being women, found that the variances in chest pain features were minimal and of such limited diagnostic value that they did not significantly aid in clinical assessment. As a result, an approach incorporating electrocardiograms and repeated high-sensitivity (hs) troponin tests is deemed the most efficacious diagnostic strategy.

The study by Meyer *et al*^1^ revealed that while the system delay has improved from 2000 to 2016 and was consistent across sexes, the total ischaemic time remained significantly longer for women. This disparity persisted nearly unchanged across the three time periods examined and was attributed to an increased delay for women in reaching the FMC.

The VIRGO Study^17^ more recently demonstrated the persistence of wait time at FMC, particularly longer wait times for some women who were at higher risk of presenting late (>6 hours after symptom onset; 35% vs 23%; p=0.002). However, Newman *et al* demonstrated that women were more likely to call emergency services during a heart attack.^18^ In the current study, we found no sex difference in the time between symptom onset and FMC. In addition, it is well-established that clinical presentation differs between sexes, with women more frequently experiencing atypical associated symptoms such as dyspnoea, weakness, fatigue and indigestion.^16 19 20^ For instance, shoulder and arm pain are twice as predictive of ACS in women than in men.^21^ Our institutional guidelines recommend an ECG and a hs troponin assay for any suspected or uncertain situation. Here, the time between FMC and REVASC is nearly identical between sexes. This is likely because our centre is a tertiary referral hospital with a well-established protocol for managing STEMI. Overall, our findings show no sex disparity in wait times for STEMI management. This is encouraging and suggests that efforts to enhance sex equity in STEMI care are having a positive impact.^22^

In a study of nearly 6000 New England residents hospitalised with AMI, Nguyen *et al* found that when care-seeking behaviour after acute coronary symptom onset has not changed significantly from 1986 to 2005, sex differences in prehospital delay have narrowed.^23^

### Access to REVASC

Our analysis included all patients undergoing percutaneous REVASC. Earlier assumptions of disparities in women’s access to reperfusion therapies are now linked more to age and comorbidities than sex. The CIAM Study, involving 1000 patients with ACS from 10 Spanish hospitals, highlighted treatment discrepancies driven by the advanced age of female patients rather than sex. Contrary to earlier findings, no significant reduction in reperfusion therapies was observed among women with STEMI. A higher incidence of microvascular diseases in women may explain the prevalence of angina despite non-significant epicardial arteries, highlighting the need for further research into these conditions.^24^

**Figure 3:**
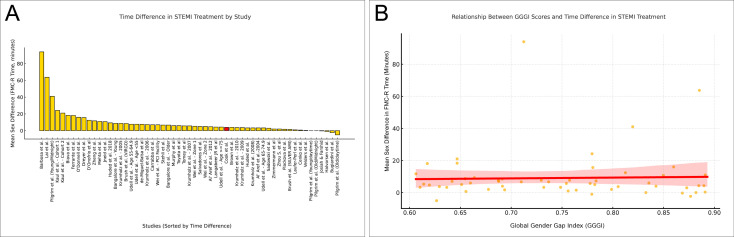
Comparison of sex differences in first medical contact-to-reperfusion (FMC-R) (or door-to-balloon) time across studies and relationship with the Global Gender Gap Index (GGGI). (A) Mean sex difference in FMC-R time across studies. Each yellow bar represents the mean time difference reported in an individual study, with positive values indicating longer delays for women compared with men. The red bar specifically represents data from the current study. (B) Relationship between the GGGI and the mean sex difference in FMC-R time. Each point represents data from a single study, with the GGGI on the x-axis and the mean time difference on the y-axis. The red line indicates the linear regression trend, with the shaded region representing the 95% CI. STEMI, ST-segment elevation myocardial infarction.

Sex differences in STEMI management are clearly reflected in FMC-R (or door-to-balloon) times. Studies^1725^,^56^ from the past two decades show this gap varies and has evolved (figure 3A). Factors include network efficiency, addressing stereotypes (eg, only men over 50 have heart attacks) and recognising that women may present atypical symptoms. Even efficient networks face challenges, such as managing young women’s heart attacks at night.^27^

Societal approaches to gender equality may also play a role. The ‘World Economic Forum’ annually measures a *Global Gender Gap Index* for each country. We explored the relationship between this index and temporal differences in care by sex, hypothesising that discrimination might delay care for the disadvantaged sex. Conversely, fully egalitarian societies should show no differences. The results, shown in figure 3B, revealed no clear correlation. This indicates that societal gender roles may not be a decisive factor in STEMI care. Instead, educational efforts—emphasising that heart attacks also occur in women and younger individuals—could improve network efficiency.^22^

### Clinical outcomes

No substantial sex-based differences in clinical outcomes were detected. Eastern European research by Cenko *et al*^57^ noted slight variances, particularly an uptick in all-cause mortality among younger women. This suggests a potential risk for young women, possibly due to restricted healthcare access and higher likelihood of missed diagnoses. However, contrasting mortality rates observed in the Dutch ACS registry become non-significant when age adjusted, diminishing perceived sex disparities.^9^

### Limitations and future research

The scope of this study was confined to live hospital arrivals diagnosed with STEMI, lacking data on clinical presentation symptoms and excluding other ACSs categories. It is possible that there were sex differences in non-STEMI syndromes. In this regard, Huber *et al*,^5^ using medical insurance coding statistics from their introduction in Switzerland, analysed data from 224 249 hospitalisations. Their findings showed that the patients studied were 8 years older than our cohort and a minority presented with STEMI (32% of women and 36% of men). In this non-specific group, the authors found a significant sex difference with a decrease in diagnostic (30% of men but 24% of women having a coronary angiogram) and therapeutic procedures (thrombolysis, PCI or coronary artery bypass graft) in favour of men.

These data might suggest that there is a failure to diagnose and treat women presenting with a heart attack in Switzerland. The alternative is that under economic pressure, medical coders have systematically assigned a diagnosis of ACS when cardiac enzymes (such as hs troponin) are elevated.

Schukraft *et al*^58^ performed a systematic follow-up of all patients presenting with elevated hs cardiac troponin T (50 ng/L) on admission, with women comprising 30% of the cohort. Final diagnoses included WHO type-1 MI in 40% (15% STEMI, 23% NSTEMI) and type-2 MI in 21%. The remaining cases (39%) were classified as myocardial injury. Combining new-onset MI (WHO class I) with other types of infarction or myocardial injury can introduce bias, especially since older patients with multiple comorbidities are more susceptible to myocardial injury during hospitalisation. In our study, we focused solely on prospective data from patients with documented STEMI, revealing no sex differences within this subgroup. However, being a single-centre study may limit the generalisability of the results. Therefore, further research is necessary to investigate sex variances across all presentations of ACSs and to elucidate the underlying reasons for observed diagnostic and therapeutic sex differences. There is also an urgent need for developing targeted interventions to enhance ACS care for women.

## Conclusion

Despite slight delays and differing risk profiles for women with STEMI, clinical outcomes are comparable between sexes. As women may present with less typical symptoms, continuous attention is necessary to ensure sex equity in the management of STEMI.

## Data Availability

All data relevant to the study are included in the article or uploaded as supplementary information.
